# Supplementary material for: A fault-intrusion-tolerant system and deadline-aware algorithm for scheduling scientific workflow in the cloud
Source: PeerJ Comput Sci. 2021 Nov 2;7:e747. doi: 10.7717/peerj-cs.747 (PMC8576554; doi:10.7717/peerj-cs.747)
Supplement: Supplemental Information 6 [file peerj-cs-07-747-s006.zip › Myproject1.1/docs/org/workflowsim/utils/Parameters.FTCMonitor.html]

Parameters.FTCMonitor


JavaScript is disabled on your browser.


- Overview
- Package
- Class
- Use
- Tree
- Deprecated
- Index
- Help

- Prev Class
- Next Class

- Frames
- No Frames

- All Classes

- Summary:
- Nested |
- Enum Constants |
- Field |
- Method

- Detail:
- Enum Constants |
- Field |
- Method


org.workflowsim.utils

## Enum Parameters.FTCMonitor

- java.lang.Object
- - java.lang.Enum<Parameters.FTCMonitor>
  - - org.workflowsim.utils.Parameters.FTCMonitor

- All Implemented Interfaces:
  :   java.io.Serializable, java.lang.Comparable<Parameters.FTCMonitor>

  Enclosing class:
  :   Parameters

  ---

    

  ```
  public static enum Parameters.FTCMonitor
  extends java.lang.Enum<Parameters.FTCMonitor>
  ```

- - ### Enum Constant Summary

    Enum Constants

    Enum Constant and Description

    `MONITOR_ALL`

    `MONITOR_JOB`

    `MONITOR_NONE`

    `MONITOR_VM`
  - ### Method Summary

    Methods

    | Modifier and Type | Method and Description |
    | --- | --- |
    | `static Parameters.FTCMonitor` | `valueOf(java.lang.String name)` Returns the enum constant of this type with the specified name. |
    | `static Parameters.FTCMonitor[]` | `values()` Returns an array containing the constants of this enum type, in the order they are declared. |

    - ### Methods inherited from class java.lang.Enum

      `clone, compareTo, equals, finalize, getDeclaringClass, hashCode, name, ordinal, toString, valueOf`
    - ### Methods inherited from class java.lang.Object

      `getClass, notify, notifyAll, wait, wait, wait`

- - ### Enum Constant Detail


    - #### MONITOR\_NONE

      ```
      public static final Parameters.FTCMonitor MONITOR_NONE
      ```


    - #### MONITOR\_ALL

      ```
      public static final Parameters.FTCMonitor MONITOR_ALL
      ```


    - #### MONITOR\_VM

      ```
      public static final Parameters.FTCMonitor MONITOR_VM
      ```


    - #### MONITOR\_JOB

      ```
      public static final Parameters.FTCMonitor MONITOR_JOB
      ```
  - ### Method Detail


    - #### values

      ```
      public static Parameters.FTCMonitor[] values()
      ```

      Returns an array containing the constants of this enum type, in
      the order they are declared. This method may be used to iterate
      over the constants as follows:

      ```
      for (Parameters.FTCMonitor c : Parameters.FTCMonitor.values())
          System.out.println(c);
      ```

      Returns:
      :   an array containing the constants of this enum type, in
          the order they are declared


    - #### valueOf

      ```
      public static Parameters.FTCMonitor valueOf(java.lang.String name)
      ```

      Returns the enum constant of this type with the specified name.
      The string must match *exactly* an identifier used to declare an
      enum constant in this type. (Extraneous whitespace characters are
      not permitted.)

      Parameters:
      :   `name` - the name of the enum constant to be returned.

      Returns:
      :   the enum constant with the specified name

      Throws:
      :   `java.lang.IllegalArgumentException` - if this enum type has no constant
          with the specified name
      :   `java.lang.NullPointerException` - if the argument is null


- Overview
- Package
- Class
- Use
- Tree
- Deprecated
- Index
- Help

- Prev Class
- Next Class

- Frames
- No Frames

- All Classes

- Summary:
- Nested |
- Enum Constants |
- Field |
- Method

- Detail:
- Enum Constants |
- Field |
- Method
